# Acetylation of Histone H3 in Cancer Progression and Prognosis

**DOI:** 10.3390/ijms252010982

**Published:** 2024-10-12

**Authors:** Paulina Miziak, Marzena Baran, Lidia Borkiewicz, Tomasz Trombik, Andrzej Stepulak

**Affiliations:** Department of Biochemistry and Molecular Biology, Medical University of Lublin, 1 Chodzki Street, 20-093 Lublin, Poland; marzenabaran@umlub.pl (M.B.); lidia.borkiewicz@umlub.pl (L.B.); tomasz.trombik@umlub.pl (T.T.)

**Keywords:** histone H3, histone acetylation, cancer, cancer progression, biomarker, cancer prognosis

## Abstract

Cancer is a multifactorial disease resulting from both genetic factors and epigenetic changes. Histone acetylation, a post-translational modification, which alters chromatin architecture and regulates gene expression is associated with cancer initiation, development and progression. Aberrations in global histone acetylation levels are observed in various cancer cells and are also associated with patients’ tumor aggressiveness. Therefore, histone acetylation may have prognostic utility and serve as a potential biomarker of cancer progression and patients’ prognosis. The reversible modification of histones by an acetyl group is versatile. One particular histone can be acetylated on different lysine residues, subsequently resulting in different biological outcomes. Here, we discuss recent findings on the acetylation of the highly conserved histone protein H3 in the context of cancer biology. Specifically, we review the acetylation of particular H3 residues in various cancer types. We further highlight the significance of H3 acetylation levels as a potential cancer biomarker with prognostic implications.

## 1. Introduction

Cancer’s complexity, with incidence and mortality still growing in many countries, makes it a life-threatening disease worldwide [1]. Clinical outcomes for cancer patients, including, for example, metastatic potential, response to chemotherapy and risk of recurrence, often differ completely among patients with lesions with the same localization, stage or histopathological and molecular cancer subtype [2,3,4]. Cancer is the first human disease to be associated with epigenetic alterations [5]. Elucidating the role of epigenetic marks such as histone acetylation and other post-translational modifications (PTMs) is key to understanding the mechanisms of cancer cell proliferation and drug resistance and the modifications in tumor microenvironment composition that favor tumor growth [6]. Given its importance in the process of carcinogenesis, histone acetylation is being explored as a molecular marker defining the oncogenic potential of different tumors together with prediction of clinical outcomes [7].

Histones are highly conserved among Eukaryotes and are positively charged nuclear proteins that form histone octamer, which acts as a linker, stabilizing the entire structure of the nucleosome [8,9,10]. Various types of histone PTMs have been identified, with prime modifications including acetylation, methylation, phosphorylation, ubiquitylation, glycosylation, ADP-ribosylation, and many more described [11,12,13,14]. The general principle relies on different chemical or protein moieties being covalently attached mainly to the histones’ amino-terminal tail domains, although they may also be present in the core of histones [15]. Histones’ PTMs are highly dynamic and reversible; they can be “written” and “erased” by structurally related enzymes [13,16]. The combination of PTMs of the specific residues of particular histones in the chromatin structure was defined as the “histone code” [17], which resulted in a hypothesis stating that gene expression could be regulated by the histones’ PTMs in a cell-type or context-specific manner [18].

Acetylation (Ac) of histone proteins is one of the first described (ca. 60 years ago) histone PTMs [19], and it plays an important structural and functional role in the nucleus. The histone as an octamer consists of two copies of four core histones, namely H3, H4, H2A and H2B, and is prone to be acetylated [15,20]. Acetylation alters chromatin structure and thus influences gene expression [21,22,23,24,25]. Dysregulation of histone acetylation is associated with cancer initiation, development and progression. Hence, enzymes involved in histone acetylation and deacetylation are popular drug targets [26,27,28].

In recent years, it has become clear that there is crosstalk between various epigenetic modifications, i.e., they act in coordination rather than independently to generate diverse functional effects. For example, key epigenetic processes that are involved in the regulation of gene expression, apart from histones’ modifications, are DNA methylation and the recruitment of non-coding RNAs. A series of these events together form a complex epigenetic landscape (recently reviewed in [29]).

The interplay between, for instance, histone acetylation and DNA methylation is crucial for the regulation of gene expression and the overall structure of chromatin. DNA methylation, which involves the addition of methyl groups to cytosine residues, often leads to gene silencing by compacting the chromatin. On the other hand, histone acetylation generally promotes a more relaxed chromatin structure, enhancing transcriptional activity. Specific DNA methylation patterns can dictate the acetylation of histones, thereby controlling the availability of transcriptional machinery at gene promoters. Conversely, acetylated histones can “attract” factors that either promote or inhibit DNA methylation, further affecting gene expression. This complex interplay is essential not only for normal cellular functioning but also for understanding diseases like cancer, where the balance between these epigenetic modifications can be disrupted, leading to uncontrolled cell growth and altered gene regulation [30,31,32]. Interestingly, it has been shown that the methylation status of DNA in somatic cells is not static; rather, it is dynamically adjusted in relation to gene activity. This regulation is facilitated by active demethylase enzymes that are influenced by histone acetylation [33].

In this review, we highlight the importance of the acetylation of highly conserved histone protein H3 in the context of cancer progression. We discuss the role of acetylation of particular lysine residues on this histone in different cancer types. The review also discusses the prognostic potential of this modification with the possibility of its use as a potential cancer biomarker. Detailed knowledge of the role of acetylation at each specific residue of histone H3 is a crucial step in deciphering the entire epigenetic landscape, which is essential for understanding cancer as a complex and multifaceted disease.

## 2. Histone Acetylation Mechanism and Enzymes Involved

**Figure 1:**
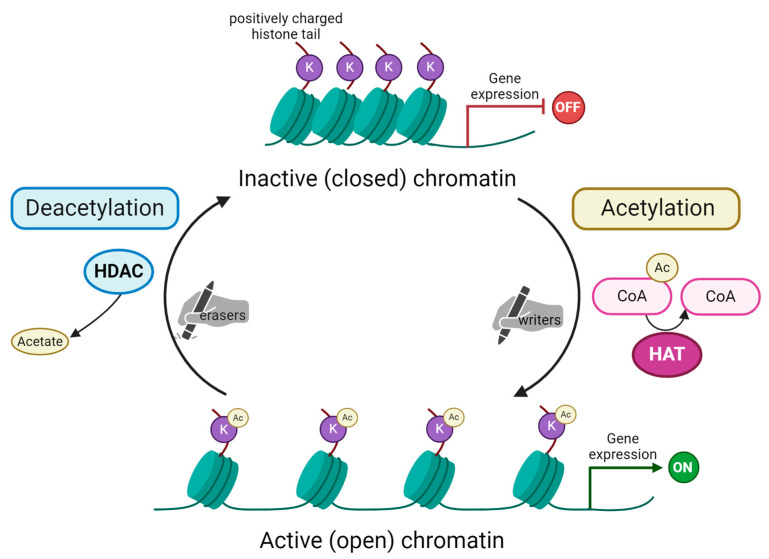
Schematic representation of histone acetylation and deacetylation process. Acetylation of lysine (K) residues on substrate histone protein is carried out by histone acetyltransferases (HAT). These enzymes are the so-called “writers” able to add acetyl groups mostly on histone tails. In the acetylation reaction, acetyl coenzyme A (here Ac-CoA) is converted into coenzyme A (CoA). Subsequently, acetylated histones impact chromatin’s structure, making it open and, as a consequence, activating gene expression. The process is reversible due to the action of histone deacetylases (HDAC), the “erasers” that remove the acetyl group from histone tails. This reaction releases acetate as a by-product. Chromatin remains in its condensed, inactive (closed) form. This blocks transcription machinery from accessing genes’ promoter regions and, in turn, represses gene expression. Created with Biorender.com.

Acetylation is catalyzed by the lysine (K) acetyltransferases family of enzymes (KATs), with many being able to modify a wide range of proteins. Histone acetylation is carried out by a specific class of KATs, known as HATs, which transfer an acetyl group from acetyl-coenzyme A (acetyl-CoA) to a specific ε-amino group of target K residues of histones (Figure 1). HATs are divided into four major families based on sequence homology, structural features and functional roles. These are as follows: GCN5 (general control non-repressed protein 5)-related N-acetyltransferases (GNAT); p300 and CBP proteins (protein of 300 kDa and CREB-binding protein); MYST (named for its members such as MOZ, Ybf2/Sas3, Sas2, and Tip60) and Rtt109 (regulator of Ty1 transposition gene production 109) [34,35]. This process can be reversed by HDACs. Histone deacetylases are classified into two groups based on catalytic mechanisms, i.e., canonical zinc-dependent (Zn^2+^-dependent) HDACs and sirtuins (SIRT) that utilize coenzyme nicotinamide adenine dinucleotide (NAD^+^) as a cofactor [36]. Specifically, the HDACs’ mammalian family has also been divided into four classes (I–IV) based on structural and functional characteristics. The division is based on the sequence similarity with the yeast enzymes. Class I of HDACs includes HDACs 1–3 and 8; class II HDACs 4–7 and 9–10; class III, also known as sirtuins, contains SIRT1–7; and class IV includes HDAC11. HDACs belonging to classes I, II and IV are Zn^2+^-dependent enzymes [37].

Both histone acetyltransferases and deacetylases are necessary for gene expression and its regulation [38]. HATs-induced gene expression is mediated by altering the spatial structure of the nucleosome, loosening the chromatin and increasing the availability of DNA for the number of sequence-specific transcription factors (TFs) [39]. In other words, acetylation within the histone tail brings modification of the lysine residues that allows the chromatin opening, due to suppressing the molecular interaction between positively charged lysine residues and negatively charged nucleosomal DNA. Acetylated lysine residues mediate the binding of many bromodomain-containing (BRD-containing) TFs and chromatin remodeling complexes (e.g., BAF) to subsequently open chromatin [40]. The family of the BRD-containing acetyl-lysine binding proteins also includes the BET (the bromodomain and extra-terminal domain) family of proteins, namely BRD2, BRD3, BRD4 and BRDT [41]. Pharmacological inhibition of BET proteins induces mitotic dysfunction, thus blocking the growth of triple-negative breast cancer (TNBC) cells [42].

The aberrant expression of HDACs in cancer made them prominent therapeutic targets. Many small-molecule HDAC inhibitors (HDIs) were developed, and they can be grouped according to their chemical structure [43]. Some of them are already accepted by the Food and Drug Administration (FDA) for the treatment of specific cancers, e.g., vorinostat for the treatment of advanced and refractory cutaneous T-cell lymphoma [44]; others are the subject of investigation in several clinical trials for other types of cancers [43]. HDIs represent a promising class of compounds in cancer therapy as other drugs, e.g., targeting receptors in solid tumors have limited efficacy as the cell may switch on alternative signaling pathways or receptor mutations may occur. HDIs act at the nuclear level where many pathways converge, though acting on specific deacetylases or selectively on the acetylation of individual lysines as presented in our previous studies [45]. Despite their potential, HDIs exhibit pleiotropic effects and can also have side effects. However, clinical trials have demonstrated manageable safety profiles for certain HDIs, making them attractive compounds for further research and development in oncology. The current information regarding clinical trials can be found at: https://clinicaltrials.gov/ or https://www.clinicaltrialsregister.eu/.

## 3. Histone H3

The way to mark post-translational modifications of histones has been standardized by the Brno nomenclature, consisting of the name of the histone, abbreviation of amino acid residues and the place and type of modification [46]. For example, H3K4ac indicates the acetylation of a lysine (K) residue at the fourth position of the H3 histone.

Histone H3 has been the most extensively modified post-translationally among all histones [47]. Thirteen acetylated K residues have been identified in human histone H3 (UniProt ID: P68431), according to the CPLM 4.0 database [48], (accessed on: January 30, 2024), including K4, K9, K14, K18, K23, K27, K36, K37, K56, K64, K79, K115 and K122 (Table 1). Most of them were reported to have an impact on patients’ outcomes in different cancer types [49]. Notably, in Eukaryotes, histone variants exist [9], with several variants for histone H3 identified in humans. These are categorized into canonical variants (H3.1 and H3.2) and replacement variants (H3.3, CENP-A, H3t, H3.X, and H3.Y). H3.1, H3.2 and H3.3 are the most prevalent variants, sharing a high degree of sequence similarity (over 96% for H3.1/H3.2 vs. H3.3, and over 99% for H3.1 vs. H3.2). These variants may, therefore, have similar PTMs on conserved amino acid residues. However, H3.1 and H3.2 are found predominantly in heterochromatin, while H3.3 is associated with euchromatin [47].

## 4. The Major H3 Modifications via Acetylation in Cancer

**Figure 2:**
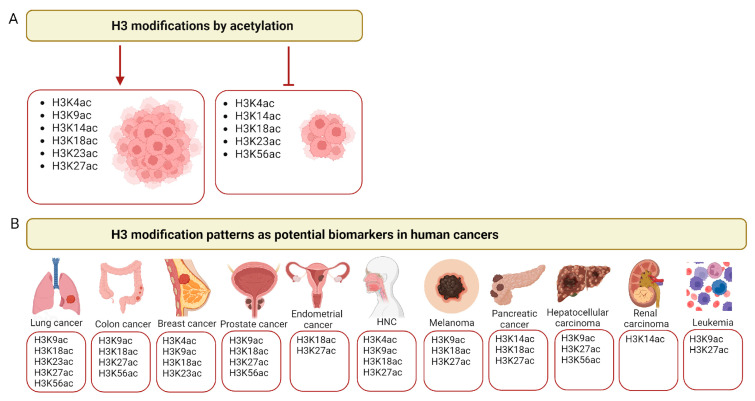
Histone H3 acetylation in cancer. (**A**) Examples of major histone H3 modifications by acetylation that promote (**left** panel) or inhibit (**right** panel) tumor growth. (**B**) Examples of the major lysines (K) acetylated in histone H3 across different cancer types that may serve as cancer biomarkers. HNCs—head and neck cancers. Created with Biorender.com.

Changes in the acetylation of histone H3 may be associated with the degree of tumor invasiveness. Depending on the type of modification, they may either be associated with stimulation or inhibition of cancer cells’ growth (Figure 2). These depend on the context of the tumor, the cell type and the acetylation site as confirmed by both experimental data in vitro and histopathological studies.

**The major H3 modifications are described below.**

### 4.1. H3K4ac

Acetylation of histone H3 has frequently been associated with the activation or inhibition of promoters of specific genes involved in the proliferation of cancer cells. A cell-type-specific pattern related to breast cancer and head and neck cancer can be observed. The increased level of H3K4ac was discovered, for instance, in the proximity to promoter regions of estrogen receptor (ER) signaling-responsive genes of MCF7 breast cancer (BC) and MDA-MB-231 TNBC cell lines (see: Table 2 summarizing the selected H3 modifications in various cancer cell lines). Particularly in MCF7 cells, increased levels of H3K4ac were observed near the promoters of genes such as *ESR1* (estrogen receptor α), *PGR* (progesterone receptor) and *GREB1* (growth regulation by estrogen in breast cancer 1), an early estrogen-responsive gene. Cell-specific patterns of H3K4ac enrichment at gene promoters were detected for genes involved in the regulation of critical proteins associated with the epithelial–mesenchymal transition (EMT). An increased level of H3K4ac was observed at the *VIM* (vimentin) promoter in MDA-MB-231 cells, while the transcription factor *GATA3* and its downstream target *FOXA1* (forkhead box F1) exhibited a decreased level of H3K4ac in MDA-MB-231 cells compared to MCF7 and MCF10A cells. Thus, H3K4ac was suggested as an epigenetic modification associated with the early stages of BC cells’ carcinogenesis [50]. In line with this, H3K4ac in BC was also identified as a target of Tat-interactive protein (TIP60) acetyltransferase (lysine acetyltransferase 5, KAT5), which belongs to the MYST acetyltransferases’ family. Studies conducted on MCF7 (ER-positive) and MDA-MB-231 (ER-negative) cells depleted of TIP60 in mice xenografts showed that TIP60 functions in BC development are ER expression-dependent [51].

H3K4ac modification has also been found in the promoter regions of EMT marker genes, such as *CDH1* (E-cadherin), *GLI1* (glioma-associated oncogene homolog 1) and *SMO* (smoothened homolog precursor) of the Hedgehog signaling pathway, which is involved in cancer cell migration and invasion, as shown in the head and neck squamous cell carcinoma (HNSCC) FaDu cell line [52]. Similarly, the progression and stemness of head and neck cancer (cancer cells and tumor) are associated with other EMT-inducing genes, such as TF *FOXF1* and *BMI1* (proto-oncogene Polycomb ring finger), which are most probably regulated by H3K4ac modification [52,53]. The cross-talk between H3K4ac and the EMT phenotype that results in cancer progression is also associated with the activity of HDACs, whose inhibition enhances H3K4ac. It has also been shown that under hypoxia, HDAC3 removes the H3K4ac and regulates the EMT marker genes’ expression. This regulation may be responsible for the hypoxia-induced EMT and metastasis [52,54].

Another study aimed to combine in vitro research with clinical observations, pointing to H3K4ac as a target of the sirtuin family of NAD^+^-dependent protein deacetylase 1 (SIRT1). Based on 135 human breast tumors compared to the matched normal tissues and five human-derived cell lines, an inverse correlation between SIRT1 and H3K4ac as well as H3K9ac and H4K16ac expression patterns was shown. As documented in recent studies, SIRT1 has also been identified as being overexpressed in breast cancer tissues, where it is associated with worse overall survival and a higher possibility for metastasis [55]. Overall, H3K4ac appears to be a target of deacetylase modifications from various phylogenetically distinct families, each employing different catalytic mechanisms. Consequently, this acetylation could represent a potential epigenetic target in breast cancer treatment. For instance, targeting HDAC1 can impact SIRT1’s function, potentially offering a novel therapeutic approach for treating breast cancer [55,56,57].

**Table 2:** An overview of the selected H3 modifications by acetylation across various cancer cell lines.

Acetylated Residues	Cancer Type/Cell Line(s)	Associated Gene(s)	Biological Significance	Ref.
H3K4ac	breast cancer (MDA-MB-231)	*TIP60*	Early stages of breast cancer transformation; initiation of EMT process; influence on estrogen response.	[50,51]
H3K4ac	head and neck squamous cell carcinoma (FaDu)	*GLI1*, *SMO*	Involvement in cancer cell migration and invasion.	[52]
H3K9ac	breast cancer (MDA-MB-231)	*TGF-β*, *SNA1*/*2*	Enhancement of TGF-β signaling; control of metastasis development.	[58]
H3K9ac and H3K27ac	acute myeloid leukemia (HL-60, ML-2, MOLM-13 and MV4-11)	*GATA2*, *TAL1*, *CEBPA*, *SPI1*	Enhancement of the expression of key transcription factors involved in differentiation therapy.	[59]
H3K14ac	renal cell carcinoma (786-O)	*PBRM1*	Tumor suppressor activity of PBRM1; enhancement of tumor growth upon dysregulation.	[60]
H3K18ac	lung cancer (A549)	*ING5*	Decreased invasiveness of cancer cells.	[61]
H3K18ac	mantle cell lymphoma (HBL-2)	*HDAC1*	Promotion of cell survival and enhancement of response to therapeutic agents.	[62]
H3K23ac	glioblastoma (LN428, LN340, U87, LN229, D54, T98G, U251 and LN444)	*TRIM24*, *KAT6B*, *PIK3CA*	Enhancement of cell proliferation and tumorigenesis.	[63]
H3K27ac	esophageal squamous cell carcinoma (Eca-109 and TE-1)	*CCAT1*	Enhancement of cancer cell proliferation.	[64]
H3K27ac	lung squamous cell carcinoma (H266, SK-MES-1)	*YAP1*	Enhancement of cancer cell proliferation.	[65]
H3K27ac	acute myeloid leukemia (MLL-AF10)	*EP300*	Promotion of cancer cell survival and proliferation.	[66]
H3K9ac H3K27ac H3K56ac	hepatocellular carcinoma (PLC-8024)	*ACACA*, *FASN*, *ACSS1*, *ACSS2*	Enhancement of cancer cell survival under hypoxic stress.	[67]

### 4.2. H3K9ac

H3K9ac is considered as a marker of active chromatin preventing K9 methylation, which is a signal for transcriptional silencing and the formation of heterochromatin [68]. For instance, in MDA-MB-231 TNBC cells, an increased level of H3K9ac was observed in a chromatin region close to the promoter of *TGFBR2* (transforming growth factor beta receptor 2) regulated by the expression of *SNAI1/2* (zinc finger proteins Snail and Slug (*SNAI1/2*). This leads to enhanced transcription of transforming growth factor β (*TGF-β*) and stimulation of the TGF-β pathway, which is a major regulator of cancer metastasis [58].

Depending on the cancer cell type, the different intensities of the H3K9ac level were demonstrated. The majority of reports present the histological expression in patients’ tissues. In prostate [69] and ovarian [70] tumors, the downregulation of H3K9ac has been linked with tumor progression that underscores the significance of epigenetic mechanisms during carcinogenesis. Quantitative immunohistochemistry with high-resolution digital image analysis (DIA) of the nuclear chromatin enabled us to distinguish between different pre-cancerous prostatic pathologies and prostate adenocarcinoma [69]. Likewise, in epithelial ovarian tumors, the decrease in H3K9ac levels was linked with the higher histological grade and the clinical stage, suggesting that H3K9ac levels might act as a potential biomarker for disease prognosis [70]. A similar study using standard immunohistochemical (IHC) examination of prostate cancer (PCA) tissues with antibodies against various histones’ modifications revealed high heterogeneity in the pattern of chromatin modifications. The analysis of histone modification patterns including H3K9ac as well as H3K18ac and H4K12ac and methylation (H3K4me2 and H4R3me2) allowed us to divide patients into two groups with varying risks of tumor recurrence, independently of other clinicopathological features. The differences in epigenetic modifications were evident in low-grade tumors; for example, in group 1, the median percent of cell staining for H3K9ac was 90%, whereas that in group 2 was 16%. These findings suggest the potential role of histone H3K9ac as an epigenetic tool for the identification of patients with different risks of PCA recurrence [26].

Similar findings were observed in patients with different histological types of tumors (squamous cell carcinoma, SCC) and localizations (oral cancer). The level of H3K9ac was lower in oral squamous cell carcinoma (OSCC) compared with premalignant lesions like oral leukoplakia (OL), a common precursor of OSCC [71]. An even more detailed recent analysis showed that H3K9ac was related to different stages of oral carcinogenesis, including low- and high-grade dysplasia [72]. On the other hand, an increase in proliferation markers such as Ki-67 and vimentin was observed from OL to OSCC, suggesting that changes in H3K9ac levels occur during oral carcinogenesis and are associated with enhanced cell proliferation and possibly also EMT (the EMT markers in OSCC were recently reviewed in: [73]). It was reflected by clinical data showing that lower H3K9ac levels in OSCC tumors resulted in lower overall survival (OS) for patients [74]. Thereby, H3K9ac could be regarded as a prognostic marker in the future. However, it requires further studies.

In contrast, in hepatocellular carcinoma (HCC), elevated levels of H3K9ac (in comparison to cirrhotic and normal liver tissue) were reported, suggesting that histone hyperacetylation participates in the pathogenesis of HCC [75]. These seem to be dependent on histological cancer type and its location. It seems that these issues are also related to the clinical outcome of the disease and patients’ fate. Dysregulation of H3K9ac, together with methylation on the same moiety (H3K9me3), is associated with patients’ prognosis. For instance, patients diagnosed with non-small cell lung cancer (NSCLC) exhibiting reduced H3K9ac levels had longer disease-free survival (DFS) and OS [76], while lower H3K9ac in gliomas was associated with worse OS and progression-free survival (PFS) [71] (see: Table 3 summarizing the selected histone H3 acetylation across the selected cancer types in the clinical context). In contrast, contradictory reports exist showing no association [77]. However, it could be related to the relatively low number of patients compared to the study of Liu et al. [71].

Acetylation of histone H3, particularly at specific H3K9 and H3K27 residues, plays a significant role in regulating gene expression during the differentiation of acute myeloid leukemia (AML) cells. The use of lysine deacetylase inhibitors, such as CM-444 and CM-1758, has been shown to enhance H3 acetylation, which promotes the expression of key transcription factors necessary for myeloid differentiation across various AML subtypes. This suggests that targeted modulation of H3 acetylation in this hematopoietic malignancy could represent a promising therapeutic strategy [59].

**Table 3:** Histone H3 acetylation across the selected cancer types in the clinical context.

Cancer Type	H3 Levels	Method(-s)	Clinical Relevance	Number of Cases/Study Cohort	Patient Classification or Outcome	Ref.
Lung cancer (NSCLC)	Decreased H3K9ac level	Immunohistochemistry (IHC)	Selecting early-stage NSCLC patients for adjuvant treatment	138	Longer disease-free survival (DFS) and overall survival (OS).	[76]
Breast cancer	Increased H3K9ac level	IHC	Potential prognostic marker	235	Shorter breast cancer-specific survival (BCSS) and shorter progression-free survival (PFS).	[78]
Breast cancer	Decreased or moderate H3K9ac, H3K18ac, H4K12ac, levels	IHC	Significant clinical value of differences across breast cancer types	880	High relative levels of global histone acetylation detected in 93% of luminal-like breast tumors associated with a favorable prognosis.	[79]
Colorectal (CRC)	Increased H3K56ac level	IHC	Significant clinical prognostic value in CRC	254	Reduced tumor recurrence; longer OS.	[80]
Epithelial ovarian cancer	Increased H3K9ac and H3K27ac levels	IHC, histological grading and clinical staging	Potential prognostic marker	365	Decreased OS and PFS.	[81]
Oral squamous cell carcinoma (OSCC)	Decreased H3K9ac levels	IHC	Potential prognostic marker	86	Lower survival rates.	[74]
Oral squamous cell carcinoma (OSCC)	Increased H3K9ac and H3K18ac levels	IHC	Aggressive clinical and pathological characteristics; potential prognostic marker	100	Patients with OSCC compared to normal oral mucosa (NOM), low-grade oral epithelial dysplasia (OED) and high-grade oral epithelial dysplasia (OED).	[72]
Pancreatic cancer	Decreased H3K18ac level	Tissue microarrays (TMAs) and IHC	Prognostic and predictive biomarker	229	Predictor of poor OS.	[82]
Pancreatic cancer	Increased H3K18ac level	IHC, patients’ clinicopathologic parameters	Potential prognostic factor	119	Poor prognosis.	[83]
Glioblastoma	Decreased H3K18ac level	IHC	Potential prognostic marker	230	Increased survival.	[71]
Glioblastoma	Decreased H3K18ac level (40% of cases)	IHC	Further investigation required	48	No association with OS.	[77]

### 4.3. H3K14ac

In contrast to H3K4ac and H3K9ac, there is a limited number of reports describing the potential role of H3K14 acetylation in cancer. It seems that elevated levels of H3K9ac-H3K14ac similar to H3K18ac may be involved in the early events of thyroid tumorigenesis. In comparison to normal tissues, patient-derived thyroid follicular adenomas and carcinomas (papillary, follicular and undifferentiated) displayed a differential expression. These modifications were suggested to be involved in the early events of thyroid tumorigenesis. On the molecular level, based on in vitro experiments, the oncoproteins RET–PTC (rearranged during transfection papillary thyroid carcinoma protein), RAS and BRAF increase histone acetylation levels, while thyroid-stimulating hormone (TSH) regulates histone acetylation in non-tumorigenic thyroid cells. This highlights the role of both neoplastic transformation and hormonal stimulation in modifying the histone acetylation patterns of thyroid cells [84].

In contrast, acetylation of H3K14 was reported as crucial for tumor suppressor functions of polybromo-1 (*PBRM1*) in the 786-O kidney cancer cell line (renal cell carcinoma) [60]. Tethering *PBRM1* to chromatin is facilitated by three bromodomains (BRD 2, 4 and 5), which collaborate to bind H3K14ac with a high affinity. Genetically introduced mutations in the BRD2 domain alone were able to impair these interactions and finally resulted in abolishing *PBRM1* molecular and anti-cancer activity [60]. However, this study performed on one cancer cell line could suggest cell-type-specific interactions and should be validated on patient-derived cancer tissues.

### 4.4. H3K18ac

The H3K18ac is considered as one of the general markers for active transcription. The modification is also important for cancer development and progression as changes in the genome-wide distribution of H3K18ac may regulate the gene expression programs that drive oncogenic transformation [82,85], although the role is not entirely defined and may have divergent effects.

In PCA, Lee and colleagues [86] found increased HAT activity, directed to specific histone H3 sites in the castrate-resistant cell line (C4-2) compared to its hormone-sensitive (LNCaP) counterpart. The progression to a resistant phenotype is accompanied by H3K18 hyperacetylation, upregulation of p300 activity, and downregulation of SIRT2 expression [86]. Selective deacetylation of H3K18ac by SIRT7 is necessary for maintaining features such as anchorage-independent growth and escape from contact inhibition of human fibrosarcoma HT1080 and osteosarcoma U2OS cancer cells in vitro [85]. The mechanism responsible for the regulation of H3K18 acetylation in lung cancer cells was described by Zhang and colleagues [61]. Analysis of protein lysine acetylation in human NSCLC A549 cells showed that inhibitor of growth 5 (ING5) protein, a candidate tumor suppressor, promotes autoacetylation of p300 HAT at several lysine residues and the activation of p300 leads to acetylation of its target proteins, including H3 at position K18 [61].

Global changes in H3K18ac indicate the molecular heterogeneity of cancers and may have a wide spectrum of clinical outcomes in cancer patients. Upregulated H3K18ac as well as H4K12ac levels in pancreatic cancer have been described as independent prognostic factors for poorer survival [83]. Moreover, low H3K18ac expression correlated positively with the pancreatic cancer stage (stage I, II) [82]. In contrast, in another study, a decreased H3K18ac level has been reported as an independent predictor of poor survival for patients with stage I and II pancreatic adenocarcinoma [82]. Other studies reported that low levels of H3K18ac have been linked with better survival for patients with glioblastoma and esophageal squamous cell carcinoma (ESCC) [71,87]. Longer PFS and OS were observed for patients with primary glioblastomas expressing lower levels of H3K18ac (<74% of tumor cells) [71]. Similarly, patients with low expression of H3K18ac in ESCCs had better survival, but this association was only significant in univariate analysis [87]. Contrarily, in breast cancer, high levels of H3K18ac were associated with a more favorable prognosis in patients. Overall, high expression of histone modifications was correlated with cancers that were positive for steroid receptors, such as the androgen receptor, estrogen receptor, and progesterone receptor, and also had upregulated E-cadherin epithelial marker, breast cancer 1 gene (BRCA1) or downregulated *p53* and human epidermal growth factor 2 (*HER-2*) genes [79]. Those findings confirm that histone modifications’ patterns show a tissue- and/or time-specific heterogeneity. Hence, they may be used for predicting patients’ outcomes in different cancer types. However, H3K18ac is highly non-specific and cannot be used as a general marker of invasiveness, due to the tumor’s histological type and its location.

The role of histone H3K18ac has also been investigated in hematopoietic malignancy–mantle cell lymphoma, revealing that alkylating agents such as bendamustine and 4-hydroperoxy-cyclophosphamide induce hyperacetylation of this specific histone residue. The findings indicate that such hyperacetylation, achieved through downregulation of SIRT7 and cleavage of HDAC3, enhances the global histone acetylation and cytotoxic effects of HDAC inhibitors like romidepsin, highlighting a potential therapeutic approach for targeting H3K18 modifications in this malignancy [62].

### 4.5. H3K23ac

In addition to its role in gene expression regulation, acetylation also plays a crucial role in signaling pathways. Acetylation can, for instance, activate the TRIM24 (tripartite motif-containing 24) protein. In glioblastoma cells, H3K23ac generated by lysine acetyltransferase 6A (KAT6A, also known as MOZ and MYST3) is associated with TRIM24. Consequently, TRIM24 promotes phosphatidylinositol-4,5-bisphosphate 3-kinase catalytic subunit alpha (PIK3CA) expression and AKT phosphorylation, leading to the activation of PI3K/AKT signaling, hence enhancing glioma cell proliferation and tumorigenesis [63]. Additionally, in estrogen receptor-driven (ER-driven) BC, the TRIM24 protein acts as a “reader” of dual histone marks within the same histone tail (H3K4me0 and H3K23ac). By binding to the chromatin and the ER, TRIM24 activates estrogen-dependent genes involved in cell proliferation and tumor development. Moreover, the expression of TRIM24 is negatively correlated with the OS of non-metastatic BC patients [88]. Furthermore, the depletion of acetyltransferase KAT6B in small cell lung cancer (SCLC) enhances cancer growth in vitro and in vivo, while its restoration inhibits cell growth and formation of metastases. The KAT6B tumor suppressor role is mediated through the acetylation of H3K23 and subsequent regulation of downstream genes [89].

### 4.6. H3K27ac

Histone acetylation may also be a signal for activation of long non-coding RNAs (lncRNAs). This plays a regulatory role in oncogenesis as reported for H3K27ac, which activates colon cancer-associated transcript-1 (*CCAT1*) lncRNA in ESCC. The gain of H3K27ac at the promoter of *CCAT1*, in ESCC cells (Eca-109) compared with human esophageal epithelial cells (HET-1A) indicates that this modification accounts for *CCAT1* dysregulation. Knocking down *CCAT1* resulted in decreased proliferation and migration of ESCC cells through the regulation of the genes linked to cell proliferation, motility and adhesion. Moreover, the expression of *CCAT1* was significantly increased and correlated with poor outcomes in ESCC [64]. Similarly, the H3K27ac-mediated activation of long intergenic non-protein coding RNA 519 (LINC00519) lncRNA was reported to promote lung squamous cell carcinoma (LSCC) progression by sponging miR-450b-5p and miR-515-5p to upregulate Yes-associated protein 1 (YAP1; transcriptional regulator) and enhance LSCC growth and metastasis [65].

The importance of H3K27ac was also revealed in a comprehensive study of the chromatin landscape of high-grade gliomas in children and young adults by Krug and colleagues [90]. The analysis of patient-derived primary cell lines expressing H3K27WT (wild type, unmodified) or H3.3K27M (mutated) revealed global loss of H3K27me3 and gain of H3K27ac in H3.3K27M-containing samples. Using tumor-derived isogenic models that bear this mutation, the authors showed that H3K27ac enrichment at repeat elements in H3.3K27M gliomas increases their expression, conferring sensitivity to epigenetic therapies for this type of tumor [90].

### 4.7. H3K36ac

Histone H3 modification of lysine at position 36 has been described as a highly conserved modification from yeast to mammals. It is found mostly on the promoters of genes transcribed by RNA-polymerase II. A protein complex termed SAGA (Spt-Ada-Gcn5 acetyltransferase) contains histone acetyltransferase (Gcn5), specifically acetylates H3K36, in vitro and is also required for this modification in vivo, suggesting its role in transcriptional regulation [91]. H3K36ac has been linked to the cell cycle. It reaches its highest levels during the S-G2 phase when homologous recombination occurs. Interestingly, another epigenetic modification of residue 36 on H3, namely H3K36me3, peaks in the G1 phase of the cell cycle and coincides with a DNA repair mechanism via non-homologous end joining (NHEJ) [92]. While the tumor-suppressive function of H3K36me3 has been recognized in several cancer types, little is still known regarding the role of H3K36ac in cancer. Recently, it was shown that a nuclear protein LOXL2 (lysyl oxidase-like 2) acts as a deacetylase, targeting the removal of acetyl groups from H3K36. Overexpression of LOXL2 decreased cancer cell proliferation and suppressed the growth of xenograft tumors in animal models. Conversely, deficiency in LOXL2 led to increased acetylation of H3K36, which in turn increased the expression of various genes associated with tumor progression. The deficiency of LOXL2 in female mice caused abnormal cell proliferation and signal transduction, leading to organ enlargement and tumor development in the uterus. Reduced nuclear expression of LOXL2 was additionally linked to poor outcomes in patients with uterine endometrial cancer [93].

### 4.8. H3K56ac

Specifically, H3K56ac, in contrast to other acetylation sites, exhibits a particularly close association with cellular metabolism. Acetylation of H3K56 is sensitive to metabolite levels in the extracellular milieu and it participates in the response to DNA damage by altering the chromatin structure [94].

In hepatocellular carcinoma (HCC), cells exposed to hypoxia (a condition, in which oxygen is deficient) upregulate acetyl-CoA synthetases (ACSS1 and ACSS2), leading to increased H3 acetylation and the expression of lipogenic genes such as *ACACA* (acetyl-CoA carboxylase alpha) and *FASN* (fatty acid synthase). This suggests that acetate serves as an epigenetic metabolite. Acetate is able to induce H3 acetylation in dose- and time-dependent manners. The increased levels of H3K9ac, H3K27ac and H3K56ac at *ACACA* and *FASN* promoters, which upregulate their expression, lead to increased lipid synthesis and enhanced cancer cell survival [67].

The study performed on different cancer cell lines showed that changes in an external environment (fresh to conditioned medium, the addition of lactate) and cell density influence the dynamics of H3K56ac in response to DNA damage caused by various genotoxic agents. H3K56ac levels after DNA damage depend on the extracellular metabolites, which impact the structure of chromatin by regulating levels of chromatin-modifying enzymes SIRT1 and SIRT6. A high level of H3K56ac is important for DNA repair because it reduces the probability of introducing the mutations associated with cancer development [95].

In NSCLC tissues, SIRT6 mRNA and protein levels were downregulated compared to normal lung tissue, and SIRT6 expression was inversely correlated with H3K56ac levels. Moreover, higher SIRT6 expression was associated with early TNM stages (I-II), negative lymph node metastases, and longer OS and metastasis-free survival (MFS) [96]. A similar relationship was observed in patients with CRC, where lower nuclear expression of H3K56ac and SIRT1 was observed in cancer compared to normal tissues. Importantly, higher nuclear levels of H4K16ac and H3K56ac were associated with better patients’ survival and a lower chance of disease recurrence. These results indicate that concomitant analysis of several markers helps to better understand the role of histone modifications in cancer development [80] and that the H3K56ac level could serve as a useful cancer biomarker.

### 4.9. H3K64ac

Di Cerbo and colleagues used nanoLC-MS/MS (nano liquid chromatography coupled to tandem mass spectrometry) to identify novel histone H3 modifications. Among these, they found H3 acetylation at lysine 64 (H3K64ac); a modification enriched in euchromatin. They showed that this modification can directly impact the stability and dynamics of nucleosomes, thereby influencing transcriptional regulation [97]. Acetylation of lysine residues 64 and 122 in the globular domain of histone H3 (H3K64ac and H3K122ac, respectively) marks active gene promoters and also a subset of enhancers [98]. The possible role of these modifications in cancer is still to be elucidated.

## 5. Discussion

It has been well documented that acetylation of highly conserved histone protein H3 influences the expression of various genes, which in turn affects cell division and proliferation depending on the cellular environment. Various studies across cancer cell line models have identified acetylation marks on histones H3 (e.g., H3K4ac, H3K9ac, H3K27ac) as being strongly associated with cancer progression, invasion and metastasis.

Acetylation at lysine 4 (K4) of H3, identified particularly in promoter regions of genes involved in the estrogen receptor signaling pathway, regulates their expression and is implicated in the progression of luminal breast cancer [50]. However, the prognostic value of this modification may be limited to breast cancer subtypes. Additionally, global levels of acetylation at other residues, e.g., H3K9ac, have also been suggested as prognostic markers in breast cancer, with increased levels linked to shorter breast cancer-specific survival (BCSS) and shorter progression-free survival (PFS). Yet, the modification may not be specific to cancer subtypes, regions of the same tumor and tumor stages.

What is more, different cancer types, including lung, breast, colorectal, bladder and prostate cancers, reveal distinct associations between histone acetylation levels and clinical outcomes. Thus, the same modifications can have different implications. In some cases, acetylation levels do not correlate with disease stage and/or clinical outcomes. These also limit the prognostic utility.

Emerging evidence has also highlighted the role of histone H3 acetylation in hematopoietic malignancies such as leukemia and lymphoma [59,62,99].

Altered acetylation patterns, particularly at H3K9 and H3K27, have been linked to the dysregulation of genes involved in hematopoiesis and immune response pathways, contributing to leukemia and lymphoma. For instance, increased H3K27ac in acute myeloid leukemia (AML) has been associated with poor prognosis and resistance to therapy. In contrast, in certain lymphomas, reduced global H3 acetylation levels correlate with better responses to histone deacetylase inhibitors, suggesting a context-dependent therapeutic opportunity.

Therefore, there is a need for a comprehensive understanding of the biological significance of H3 acetylation across different cancer types and stages. It is also important to not only consider the levels of these modifications but also the complete information on the enzymes involved in specifically acetylating and deacetylating various lysine residues on H3. While H3 acetylation levels serve as strong indicators of cancer progression from initiation to aggressive metastatic phenotypes, further research is necessary to validate these modifications as reliable prognostic markers and therapeutic targets. Last but not least, accurate measurement and quantification of histone modification levels pose significant challenges and limitations in their immediate application in clinical settings.

## 6. Conclusions and Perspectives

Histone H3 acetylation plays a pivotal role in gene expression regulation, DNA damage repair and modulation of cellular signaling or protein–protein interactions, affecting cell division and proliferation in various cancer types, including breast, lung, colorectal, bladder and prostate cancers, as a consequence. Specific acetylation marks on histone H3 have emerged as strong indicators of cancer progression, invasion and metastasis. However, the prognostic value of these modifications can vary significantly across different cancer subtypes and stages, reflecting the complexity and heterogeneity of cancers. This, in turn, complicates the use of acetylation patterns as prognostic markers and therapeutic targets.

To advance the field, future research should focus on some key aspects. Firstly, there is a need for comprehensive studies to identify novel acetylation profiles and understand their in-depth role in cancer progression across diverse cancer types. This research could reveal new biomarkers for early detection and prognosis and even help in treatment response prediction. Additionally, improved methodologies for measuring and quantifying histone acetylation levels are essential to translate the findings into clinical practice. Lastly, understanding the specific enzymes involved in acetylation and deacetylation processes can provide insights into the regulatory mechanisms, thus facilitating the development of targeted therapies. Advancing knowledge in these areas holds promise for enhancing cancer management, ultimately improving patients’ outcomes.

## Figures and Tables

**Table 1:** Acetylated lysines (K) with flanking sequences in histone H3 (UniProt ID: P68431). Flanking sequences retrieved from the CPLM 4.0 database [48], accessed on 30 January 2024.

Position of Acetylated Lysine (K) in H3	Flanking Sequences
4	----ART**K**QTARKST
9	RTKQTAR**K**STGGKAP
14	ARKSTGG**K**APRKQLA
18	TGGKAPR**K**QLATKAA
23	PRKQLAT**K**AARKSAP
27	LATKAAR**K**SAPATGG
36	APATGGV**K**KPHRYRP
37	PATGGVK**K**PHRYRPG
56	REIRRYQ**K**STELLIR
64	STELLIR**K**LPFQRLV
79	REIAQDF**K**TDLRFQS
115	NLCAIHA**K**RVTIMPK
122	KRVTIMP**K**DIQLARR

## Data Availability

No data were used for the research described in the article.

## Abbreviations

Ac	acetylation
BC	breast cancer
BCSS	breast cancer-specific survival
BET	bromodomain and extra-terminal domain
BRD	bromodomain
CBP	CREB-binding protein
ChIP	chromatin immunoprecipitation
DDR	DNA damage response
DFS	disease-free survival
DIA	digital image analysis
ER	estrogen receptor
ESCC	esophageal squamous cell carcinoma
EMT	epithelial–mesenchymal transition
FDA	The Food and Drug Administration
HAT	histone acetyltransferase
HBV	hepatitis B virus
HCC	hepatocellular carcinoma
HDAC	histone deacetylase
HDI	histone deacetylase inhibitor
HNC	head and neck cancer
HNSCC	head and neck squamous cell carcinoma
IHC	immunohistochemistry
ING5	inhibitor of growth 5
K	lysine
KAT	lysine acetyltransferase
LSCC	lung squamous cell carcinoma
Me3	trimethylation
MLL1	mixed lineage leukemia 1
MFS	metastasis-free survival
NAD	nicotinamide adenine dinucleotide
NSCLC	non-small cell lung cancer
NSCLC	non-small cell lung cancer
OL	oral leukoplakia
OS	overall survival
OSCC	oral squamous cell carcinoma
PCA	prostate cancer
PFS	progression-free survival
PSA	prostate-specific antigen
PTMs	post-translational modifications
RB	retinoblastoma
SCLC	small cell lung cancer
SIRT	sirtuin
TF	transcription factor
TIP60	TAT-INTERACTIVE PROTEIN
TNBC	triple-negative breast cancer

## References

[1] Yang Y., Zhang M., Wang Y. (2022). The Roles of Histone Modifications in Tumorigenesis and Associated Inhibitors in Cancer Therapy. J. Natl. Cancer Cent..

[2] Choi H.C.W., Lam K.-O., Pang H.H.M., Tsang S.K.C., Ngan R.K.C., Lee A.W.M. (2019). Global Comparison of Cancer Outcomes: Standardization and Correlation with Healthcare Expenditures. BMC Public Health.

[3] Wang R., Zhu Y., Liu X., Liao X., He J., Niu L. (2019). The Clinicopathological Features and Survival Outcomes of Patients with Different Metastatic Sites in Stage IV Breast Cancer. BMC Cancer.

[4] Kerrigan K., Patel S.B., Haaland B., Ose D., Weinberg Chalmers A., Haydell T., Meropol N.J., Akerley W. (2020). Prognostic Significance of Patient-Reported Outcomes in Cancer. JCO Oncol. Pract..

[5] Feinberg A.P., Vogelstein B. (1983). Hypomethylation Distinguishes Genes of Some Human Cancers from Their Normal Counterparts. Nature.

[6] Lodewijk I., Nunes S.P., Henrique R., Jerónimo C., Dueñas M., Paramio J.M. (2021). Tackling Tumor Microenvironment through Epigenetic Tools to Improve Cancer Immunotherapy. Clin. Epigenet..

[7] Karim M.F., Kabir Y., Rezaei N. (2022). Role of Histone Modifications in the Progression of Cancer. Handbook of Cancer and Immunology.

[8] Park Y.-J., Luger K. (2008). Histone Chaperones in Nucleosome Eviction and Histone Exchange. Curr. Opin. Struct. Biol..

[9] Talbert P.B., Henikoff S. (2010). Histone Variants—Ancient Wrap Artists of the Epigenome. Nat. Rev. Mol. Cell Biol..

[10] Cutter A.R., Hayes J.J. (2015). A Brief Review of Nucleosome Structure. FEBS Lett..

[11] Arnaudo A.M., Garcia B.A. (2013). Proteomic Characterization of Novel Histone Post-Translational Modifications. Epigenet. Chromatin.

[12] Huang H., Lin S., Garcia B.A., Zhao Y. (2015). Quantitative Proteomic Analysis of Histone Modifications. Chem. Rev..

[13] Millán-Zambrano G., Burton A., Bannister A.J., Schneider R. (2022). Histone Post-Translational Modifications—Cause and Consequence of Genome Function. Nat. Rev. Genet..

[14] Liu R., Wu J., Guo H., Yao W., Li S., Lu Y., Jia Y., Liang X., Tang J., Zhang H. (2023). Post-Translational Modifications of Histones: Mechanisms, Biological Functions, and Therapeutic Targets. MedComm.

[15] Tessarz P., Kouzarides T. (2014). Histone Core Modifications Regulating Nucleosome Structure and Dynamics. Nat. Rev. Mol. Cell Biol..

[16] Lehtomaki E., Mackay J.P., Roberts G.C.K. (2013). Post-Translational Modification of Histone Proteins. Encyclopedia of Biophysics.

[17] Jenuwein T., Allis C.D. (2001). Translating the Histone Code. Science.

[18] Lam K.-W.G., Brick K., Cheng G., Pratto F., Camerini-Otero R.D. (2019). Cell-Type-Specific Genomics Reveals Histone Modification Dynamics in Mammalian Meiosis. Nat. Commun..

[19] Allfrey V.G., Faulkner R., Mirsky A.E. (1964). Acetylation and Methylation of Histones and Their Possible Role in the Regulation of RNA Synthesis. Proc. Natl. Acad. Sci. USA.

[20] Yang J., Song C., Zhan X. (2022). The Role of Protein Acetylation in Carcinogenesis and Targeted Drug Discovery. Front. Endocrinol..

[21] Grunstein M. (1997). Histone Acetylation in Chromatin Structure and Transcription. Nature.

[22] Turner B.M. (2000). Histone Acetylation and an Epigenetic Code. Bioessays.

[23] Baylin S.B., Jones P.A. (2016). Epigenetic Determinants of Cancer. Cold Spring Harb. Perspect. Biol..

[24] Yen C.-Y., Huang H.-W., Shu C.-W., Hou M.-F., Yuan S.-S.F., Wang H.-R., Chang Y.-T., Farooqi A.A., Tang J.-Y., Chang H.-W. (2016). DNA Methylation, Histone Acetylation and Methylation of Epigenetic Modifications as a Therapeutic Approach for Cancers. Cancer Lett..

[25] Joseph F.M., Young N.L. (2023). Histone Variant-Specific Post-Translational Modifications. Semin. Cell Dev. Biol..

[26] Seligson D.B., Horvath S., Shi T., Yu H., Tze S., Grunstein M., Kurdistani S.K. (2005). Global Histone Modification Patterns Predict Risk of Prostate Cancer Recurrence. Nature.

[27] Audia J.E., Campbell R.M. (2016). Histone Modifications and Cancer. Cold Spring Harb. Perspect. Biol..

[28] Patra S., Panigrahi D.P., Praharaj P.P., Bhol C.S., Mahapatra K.K., Mishra S.R., Behera B.P., Jena M., Bhutia S.K. (2019). Dysregulation of Histone Deacetylases in Carcinogenesis and Tumor Progression: A Possible Link to Apoptosis and Autophagy. Cell. Mol. Life Sci..

[29] Manna S., Mishra J., Baral T., Kirtana R., Nandi P., Roy A., Chakraborty S., Niharika, Patra S.K. (2023). Epigenetic Signaling and Crosstalk in Regulation of Gene Expression and Disease Progression. Epigenomics.

[30] Vaissière T., Sawan C., Herceg Z. (2008). Epigenetic Interplay between Histone Modifications and DNA Methylation in Gene Silencing. Mutat. Res..

[31] Tao L., Zhou Y., Luo Y., Qiu J., Xiao Y., Zou J., Zhang Y., Liu X., Yang X., Gou K. (2024). Epigenetic Regulation in Cancer Therapy: From Mechanisms to Clinical Advances. MedComm–Oncology.

[32] Miller J.L., Grant P.A. (2013). The Role of DNA Methylation and Histone Modifications in Transcriptional Regulation in Humans. Subcell. Biochem..

[33] Cervoni N., Szyf M. (2001). Demethylase Activity Is Directed by Histone Acetylation. J. Biol. Chem..

[34] Poziello A., Nebbioso A., Stunnenberg H.G., Martens J.H.A., Carafa V., Altucci L. (2021). Recent Insights into Histone Acetyltransferase-1: Biological Function and Involvement in Pathogenesis. Epigenetics.

[35] Sabnis R.W. (2021). Novel Histone Acetyltransferase (HAT) Inhibitors for Treating Diseases. ACS Med. Chem. Lett..

[36] Di Cerbo V., Schneider R. (2013). Cancers with Wrong HATs: The Impact of Acetylation. Brief. Funct. Genom..

[37] Park S.-Y., Kim J.-S. (2020). A Short Guide to Histone Deacetylases Including Recent Progress on Class II Enzymes. Exp. Mol. Med..

[38] Wang Z., Zang C., Cui K., Schones D.E., Barski A., Peng W., Zhao K. (2009). Genome-Wide Mapping of HATs and HDACs Reveals Distinct Functions in Active and Inactive Genes. Cell.

[39] Sterner D.E., Berger S.L. (2000). Acetylation of Histones and Transcription-Related Factors. Microbiol. Mol. Biol. Rev..

[40] Varga J., Kube M., Luck K., Schick S. (2021). The BAF Chromatin Remodeling Complexes: Structure, Function, and Synthetic Lethalities. Biochem. Soc. Trans..

[41] Wang N., Wu R., Tang D., Kang R. (2021). The BET Family in Immunity and Disease. Signal Transduct. Target. Ther..

[42] Brancato J.M., Gayle S., Weber-Bonk K., Summers M., Bebek G., Keri R. (2016). Abstract 4647: BET Protein Inhibition Blocks Growth of Triple-Negative Breast Cancer by Inducing Mitotic and Cytokinetic Dysfunction. Cancer Res..

[43] Bondarev A.D., Attwood M.M., Jonsson J., Chubarev V.N., Tarasov V.V., Schiöth H.B. (2021). Recent Developments of HDAC Inhibitors: Emerging Indications and Novel Molecules. Br. J. Clin. Pharmacol..

[44] Mann B.S., Johnson J.R., Cohen M.H., Justice R., Pazdur R. (2007). FDA Approval Summary: Vorinostat for Treatment of Advanced Primary Cutaneous T-Cell Lymphoma. Oncologist.

[45] Gumbarewicz E., Luszczki J.J., Wawruszak A., Dmoszynska-Graniczka M., Grabarska A.J., Jarząb A.M., Polberg K., Stepulak A. (2016). Isobolographic Analysis Demonstrates Additive Effect of Cisplatin and HDIs Combined Treatment Augmenting Their Anti-Cancer Activity in Lung Cancer Cell Lines. Am. J. Cancer Res..

[46] Turner B.M. (2005). Reading Signals on the Nucleosome with a New Nomenclature for Modified Histones. Nat. Struct. Mol. Biol..

[47] Xu Y.-M., Du J.-Y., Lau A.T.Y. (2014). Posttranslational Modifications of Human Histone H3: An Update. Proteomics.

[48] Zhang W., Tan X., Lin S., Gou Y., Han C., Zhang C., Ning W., Wang C., Xue Y. (2022). CPLM 4.0: An Updated Database with Rich Annotations for Protein Lysine Modifications. Nucleic Acids Res..

[49] Barnes C.E., English D.M., Cowley S.M. (2019). Acetylation & Co: An Expanding Repertoire of Histone Acylations Regulates Chromatin and Transcription. Essays Biochem..

[50] Messier T.L., Gordon J.A.R., Boyd J.R., Tye C.E., Browne G., Stein J.L., Lian J.B., Stein G.S. (2016). Histone H3 Lysine 4 Acetylation and Methylation Dynamics Define Breast Cancer Subtypes. Oncotarget.

[51] Judes G., Dubois L., Rifaï K., Idrissou M., Mishellany F., Pajon A., Besse S., Daures M., Degoul F., Bignon Y.-J. (2018). TIP60: An Actor in Acetylation of H3K4 and Tumor Development in Breast Cancer. Epigenomics.

[52] Wang J.-Q., Yan F.-Q., Wang L.-H., Yin W.-J., Chang T.-Y., Liu J.-P., Wu K.-J. (2020). Identification of New Hypoxia-Regulated Epithelial-Mesenchymal Transition Marker Genes Labeled by H3K4 Acetylation. Genes Chromosomes Cancer.

[53] Yang M.-H., Hsu D.S.-S., Wang H.-W., Wang H.-J., Lan H.-Y., Yang W.-H., Huang C.-H., Kao S.-Y., Tzeng C.-H., Tai S.-K. (2010). Bmi1 Is Essential in Twist1-Induced Epithelial-Mesenchymal Transition. Nat. Cell Biol..

[54] Lin Y.-T., Wu K.-J. (2020). Epigenetic Regulation of Epithelial-Mesenchymal Transition: Focusing on Hypoxia and TGF-β Signaling. J. Biomed. Sci..

[55] Parija M., Prakash S., Krishna B.M., Dash S., Mishra S.K. (2024). SIRT1 Mediates Breast Cancer Development and Tumorigenesis Controlled by Estrogen-Related Receptor β. Breast Cancer.

[56] Rifaï K., Judes G., Idrissou M., Daures M., Bignon Y.-J., Penault-Llorca F., Bernard-Gallon D. (2018). SIRT1-Dependent Epigenetic Regulation of H3 and H4 Histone Acetylation in Human Breast Cancer. Oncotarget.

[57] Salih A.I., Al-Sudani B.T., Mshimesh B.A.-R. (2024). Targeting POLD1 to Suppress the Proliferation and Migration of Breast Cancer MDA-MB-231 Cell Lines by Downregulation of SIRT1. Toxicol. Res..

[58] Dhasarathy A., Phadke D., Mav D., Shah R.R., Wade P.A. (2011). The Transcription Factors Snail and Slug Activate the Transforming Growth Factor-Beta Signaling Pathway in Breast Cancer. PLoS ONE.

[59] San José-Enériz E., Gimenez-Camino N., Rabal O., Garate L., Miranda E., Gómez-Echarte N., García F., Charalampopoulou S., Sáez E., Vilas-Zornoza A. (2024). Epigenetic-Based Differentiation Therapy for Acute Myeloid Leukemia. Nat. Commun..

[60] Liao L., Alicea-Velázquez N.L., Langbein L., Niu X., Cai W., Cho E.-A., Zhang M., Greer C.B., Yan Q., Cosgrove M.S. (2019). High Affinity Binding of H3K14ac through Collaboration of Bromodomains 2, 4 and 5 Is Critical for the Molecular and Tumor Suppressor Functions of PBRM1. Mol. Oncol..

[61] Zhang T., Meng J., Liu X., Zhang X., Peng X., Cheng Z., Zhang F. (2018). ING5 Differentially Regulates Protein Lysine Acetylation and Promotes p300 Autoacetylation. Oncotarget.

[62] Hiraoka N., Kikuchi J., Koyama D., Wada T., Mori S., Nakamura Y., Furukawa Y. (2013). Alkylating Agents Induce Histone H3K18 Hyperacetylation and Potentiate HDAC Inhibitor-Mediated Global Histone Acetylation and Cytotoxicity in Mantle Cell Lymphoma. Blood Cancer J..

[63] Lv D., Jia F., Hou Y., Sang Y., Alvarez A.A., Zhang W., Gao W.-Q., Hu B., Cheng S.-Y., Ge J. (2017). Histone Acetyltransferase KAT6A Upregulates PI3K/AKT Signaling through TRIM24 Binding. Cancer Res..

[64] Zhang E., Han L., Yin D., He X., Hong L., Si X., Qiu M., Xu T., De W., Xu L. (2017). H3K27 Acetylation Activated-Long Non-Coding RNA CCAT1 Affects Cell Proliferation and Migration by Regulating SPRY4 and HOXB13 Expression in Esophageal Squamous Cell Carcinoma. Nucleic Acids Res..

[65] Ye P., Lv X., Aizemaiti R., Cheng J., Xia P., Di M. (2020). H3K27ac-Activated LINC00519 Promotes Lung Squamous Cell Carcinoma Progression by Targeting miR-450b-5p/miR-515-5p/YAP1 Axis. Cell Prolif..

[66] Pan F., Iwasaki M., Wu W., Jiang Y., Yang X., Zhu L., Zhao Z., Cleary M.L. (2023). Enhancer Remodeling Drives MLL Oncogene-Dependent Transcriptional Dysregulation in Leukemia Stem Cells. Blood Adv..

[67] Gao X., Lin S.-H., Ren F., Li J.-T., Chen J.-J., Yao C.-B., Yang H.-B., Jiang S.-X., Yan G.-Q., Wang D. (2016). Acetate Functions as an Epigenetic Metabolite to Promote Lipid Synthesis under Hypoxia. Nat. Commun..

[68] Füllgrabe J., Kavanagh E., Joseph B. (2011). Histone Onco-Modifications. Oncogene.

[69] Mohamed M.A., Greif P.A., Diamond J., Sharaf O., Maxwell P., Montironi R., Young R.A.M., Hamilton P.W. (2007). Epigenetic Events, Remodelling Enzymes and Their Relationship to Chromatin Organization in Prostatic Intraepithelial Neoplasia and Prostatic Adenocarcinoma. BJU Int..

[70] Zhen L., Gui-lan L., Ping Y., Jin H., Ya-li W. (2010). The Expression of H3K9Ac, H3K14Ac, and H4K20TriMe in Epithelial Ovarian Tumors and the Clinical Significance. Int. J. Gynecol. Cancer.

[71] Liu B.-L., Cheng J.-X., Zhang X., Wang R., Zhang W., Lin H., Xiao X., Cai S., Chen X.-Y., Cheng H. (2010). Global Histone Modification Patterns as Prognostic Markers to Classify Glioma Patients. Cancer Epidemiol. Biomark. Prev..

[72] Tachaveeraphong W., Phattarataratip E. (2024). The Significance of Modified Histone H3 in Epithelial Dysplasia and Oral Cancer. Int. Dent. J..

[73] Pawlicka M., Gumbarewicz E., Błaszczak E., Stepulak A. (2024). Transcription Factors and Markers Related to Epithelial-Mesenchymal Transition and Their Role in Resistance to Therapies in Head and Neck Cancers. Cancers.

[74] Webber L.P., Wagner V.P., Curra M., Vargas P.A., Meurer L., Carrard V.C., Squarize C.H., Castilho R.M., Martins M.D. (2017). Hypoacetylation of Acetyl-Histone H3 (H3K9ac) as Marker of Poor Prognosis in Oral Cancer. Histopathology.

[75] Bai X., Wu L., Liang T., Liu Z., Li J., Li D., Xie H., Yin S., Yu J., Lin Q. (2008). Overexpression of Myocyte Enhancer Factor 2 and Histone Hyperacetylation in Hepatocellular Carcinoma. J. Cancer Res. Clin. Oncol..

[76] Barlési F., Giaccone G., Gallegos-Ruiz M.I., Loundou A., Span S.W., Lefesvre P., Kruyt F.A.E., Rodriguez J.A. (2007). Global Histone Modifications Predict Prognosis of Resected Non Small-Cell Lung Cancer. J. Clin. Oncol..

[77] Archana B., D’Cruze L., Sundaram S., Ramanathan K., Ganesh K. (2024). Immunohistochemical Expression of Histone Modification Pattern in Adult Glioblastoma. J. Cancer Res. Ther..

[78] Berger L., Kolben T., Meister S., Kolben T.M., Schmoeckel E., Mayr D., Mahner S., Jeschke U., Ditsch N., Beyer S. (2020). Expression of H3K4me3 and H3K9ac in Breast Cancer. J. Cancer Res. Clin. Oncol..

[79] Elsheikh S.E., Green A.R., Rakha E.A., Powe D.G., Ahmed R.A., Collins H.M., Soria D., Garibaldi J.M., Paish C.E., Ammar A.A. (2009). Global Histone Modifications in Breast Cancer Correlate with Tumor Phenotypes, Prognostic Factors, and Patient Outcome. Cancer Res..

[80] Benard A., Goossens-Beumer I.J., van Hoesel A.Q., Horati H., de Graaf W., Putter H., Zeestraten E.C.M., Liefers G.-J., van de Velde C.J.H., Kuppen P.J.K. (2015). Nuclear Expression of Histone Deacetylases and Their Histone Modifications Predicts Clinical Outcome in Colorectal Cancer. Histopathology.

[81] Bauer T.L., Collmar K., Kaltofen T., Loeffler A.-K., Decker L., Mueller J., Pinter S., Eisler S.A., Mahner S., Fraungruber P. (2021). Functional Analysis of Non-Genetic Resistance to Platinum in Epithelial Ovarian Cancer Reveals a Role for the MBD3-NuRD Complex in Resistance Development. Cancers.

[82] Manuyakorn A., Paulus R., Farrell J., Dawson N.A., Tze S., Cheung-Lau G., Hines O.J., Reber H., Seligson D.B., Horvath S. (2010). Cellular Histone Modification Patterns Predict Prognosis and Treatment Response in Resectable Pancreatic Adenocarcinoma: Results from RTOG 9704. J. Clin. Oncol..

[83] Juliano C.N., Izetti P., Pereira M.P., Dos Santos A.P., Bravosi C.P., Abujamra A.L., Prolla P.A., Osvaldt A.B., Edelweiss M.I.A. (2016). H4K12 and H3K18 Acetylation Associates With Poor Prognosis in Pancreatic Cancer. Appl. Immunohistochem. Mol. Morphol..

[84] Puppin C., Passon N., Lavarone E., Di Loreto C., Frasca F., Vella V., Vigneri R., Damante G. (2011). Levels of Histone Acetylation in Thyroid Tumors. Biochem. Biophys. Res. Commun..

[85] Barber M.F., Michishita-Kioi E., Xi Y., Tasselli L., Kioi M., Moqtaderi Z., Tennen R.I., Paredes S., Young N.L., Chen K. (2012). SIRT7 Links H3K18 Deacetylation to Maintenance of Oncogenic Transformation. Nature.

[86] Lee J.-H., Yang B., Lindahl A.J., Damaschke N., Boersma M.D., Huang W., Corey E., Jarrard D.F., Denu J.M. (2017). Identifying Dysregulated Epigenetic Enzyme Activity in Castrate-Resistant Prostate Cancer Development. ACS Chem. Biol..

[87] Tzao C., Tung H.-J., Jin J.-S., Sun G.-H., Hsu H.-S., Chen B.-H., Yu C.-P., Lee S.-C. (2009). Prognostic Significance of Global Histone Modifications in Resected Squamous Cell Carcinoma of the Esophagus. Mod. Pathol..

[88] Tsai W.-W., Wang Z., Yiu T.T., Akdemir K.C., Xia W., Winter S., Tsai C.-Y., Shi X., Schwarzer D., Plunkett W. (2010). TRIM24 Links a Non-Canonical Histone Signature to Breast Cancer. Nature.

[89] Simó-Riudalbas L., Pérez-Salvia M., Setien F., Villanueva A., Moutinho C., Martínez-Cardús A., Moran S., Berdasco M., Gomez A., Vidal E. (2015). KAT6B Is a Tumor Suppressor Histone H3 Lysine 23 Acetyltransferase Undergoing Genomic Loss in Small Cell Lung Cancer. Cancer Res..

[90] Krug B., De Jay N., Harutyunyan A.S., Deshmukh S., Marchione D.M., Guilhamon P., Bertrand K.C., Mikael L.G., McConechy M.K., Chen C.C.L. (2019). Pervasive H3K27 Acetylation Leads to ERV Expression and a Therapeutic Vulnerability in H3K27M Gliomas. Cancer Cell.

[91] Morris S.A., Rao B., Garcia B.A., Hake S.B., Diaz R.L., Shabanowitz J., Hunt D.F., Allis C.D., Lieb J.D., Strahl B.D. (2007). Identification of Histone H3 Lysine 36 Acetylation as a Highly Conserved Histone Modification*. J. Biol. Chem..

[92] Pai C.-C., Deegan R.S., Subramanian L., Gal C., Sarkar S., Blaikley E.J., Walker C., Hulme L., Bernhard E., Codlin S. (2014). A Histone H3K36 Chromatin Switch Coordinates DNA Double-Strand Break Repair Pathway Choice. Nat. Commun..

[93] Lu X., Xin D.E., Du J.K., Zou Q.C., Wu Q., Zhang Y.S., Deng W., Yue J., Fan X.S., Zeng Y. (2022). Loss of LOXL2 Promotes Uterine Hypertrophy and Tumor Progression by Enhancing H3K36ac-Dependent Gene Expression. Cancer Res..

[94] Vadla R., Chatterjee N., Haldar D. (2020). Cellular Environment Controls the Dynamics of Histone H3 Lysine 56 Acetylation in Response to DNA Damage in Mammalian Cells. J. Biosci..

[95] Aricthota S., Rana P.P., Haldar D. (2022). Histone Acetylation Dynamics in Repair of DNA Double-Strand Breaks. Front. Genet..

[96] Zhu B., Yan Y., Shao B., Tian L., Zhou W. (2018). Downregulation of SIRT6 Is Associated with Poor Prognosis in Patients with Non-Small Cell Lung Cancer. J. Int. Med. Res..

[97] Di Cerbo V., Mohn F., Ryan D.P., Montellier E., Kacem S., Tropberger P., Kallis E., Holzner M., Hoerner L., Feldmann A. (2014). Acetylation of Histone H3 at Lysine 64 Regulates Nucleosome Dynamics and Facilitates Transcription. eLife.

[98] Pradeepa M.M., Grimes G.R., Kumar Y., Olley G., Taylor G.C.A., Schneider R., Bickmore W.A. (2016). Histone H3 Globular Domain Acetylation Identifies a New Class of Enhancers. Nat. Genet..

[99] Fernández-Serrano M., Winkler R., Santos J.C., Le Pannérer M.-M., Buschbeck M., Roué G. (2021). Histone Modifications and Their Targeting in Lymphoid Malignancies. Int. J. Mol. Sci..

